# Pre-diagnosis alcohol consumption and mortality risk among black women and white women with invasive breast cancer

**DOI:** 10.1186/s12885-019-5991-8

**Published:** 2019-08-13

**Authors:** Huiyan Ma, Kathleen E. Malone, Jill A. McDonald, Polly A. Marchbanks, Giske Ursin, Brian L. Strom, Michael S. Simon, Jane Sullivan-Halley, Leslie Bernstein, Yani Lu

**Affiliations:** 1 Department of Population Sciences, Beckman Research Institute, City of Hope, 1500 East Duarte Rd, Duarte, CA 91010 USA; 2 Division of Public Health Sciences, Fred Hutchinson Cancer Research Center, Seattle, WA 98109 USA; 3 College of Health and Social Services, New Mexico State University, Las Cruces, NM 88003 USA; 4 Atlanta, USA; 5 Cancer Registry of Norway, Oslo Norway and Department of Nutrition, Institute of Basic Medical Sciences, University of Oslo, Oslo, Norway; 6 Rutgers, the State University of New Jersey, Newark, NJ USA; 7 Karmanos Cancer Institute, Department of Oncology, Wayne State University, Detroit, MI 48201 USA

**Keywords:** Alcohol, Wine, Beer, Liquor, Breast cancer, Mortality, White women, Black women

## Abstract

**Background:**

Alcohol consumption is associated with increased risk of breast cancer; however, its association with subsequent risk of breast cancer death is unclear.

**Methods:**

We followed 4523 women with complete information on relevant risk factors for mortality; these women were 35 to 64 years of age when diagnosed with incident invasive breast cancer between 1994 and 1998. During follow up (median, 8.6 years), 1055 women died; 824 died from breast cancer. The information on alcohol consumption before diagnosis was collected shortly after breast cancer diagnosis (average: 5.1 months) during an in-person interview which used a structured questionnaire. Multivariable Cox proportional hazards regression models provided hazard ratios (HRs) and 95% confidence intervals (CIs) for breast cancer-specific mortality, mortality due to causes other than breast cancer, and all-cause mortality associated with alcohol consumption from age 15 years until breast cancer diagnosis and during recent periods of time prior to breast cancer diagnosis.

**Results:**

Average weekly alcohol consumption from age 15 years until breast cancer diagnosis was inversely associated with breast cancer-specific mortality (*P*_trend_ = 0.01). Compared to non-drinkers, women in the highest average weekly alcohol consumption category (≥7 drinks/week) had 25% lower risk of breast cancer-specific mortality (HR = 0.75, 95% CI = 0.56–1.00). Breast cancer mortality risk was also reduced among women in the highest average weekly alcohol consumption category in two recent time periods (5-year period ending 2-years prior to breast cancer diagnosis, HR = 0.74, 95% CI = 0.57–0.95; 2-year period immediately prior to breast cancer diagnosis: HR = 0.73, 95% CI = 0.56–0.95). Furthermore, analyses of average weekly alcohol consumption by beverage type from age 15 years until breast cancer diagnosis suggested that wine consumption was inversely associated with breast cancer-specific mortality risk (wine *P*_trend_ = 0.06, beer *P*_trend_ = 0.24, liquor *P*_trend_ = 0.74). No association with any of these alcohol consumption variables was observed for mortality risk due to causes other than breast cancer.

**Conclusions:**

Overall, we found no evidence that alcohol consumption before breast cancer diagnosis increases subsequent risk of death from breast cancer.

## Background

Alcohol consumption is associated with increased risk of breast cancer [1–5]. It may also influence tumor progression and breast cancer recurrence, thus affecting risk of breast cancer-specific mortality. Previous findings regarding the association of pre-diagnosis alcohol consumption with risk of breast cancer-specific mortality are mixed, showing decreased risk [6–8], increased risk [9–11], and no association [12–20]. A meta-analysis of 11 published studies demonstrated that moderate pre-diagnosis alcohol consumption was associated with reduced risk of all-cause mortality, but did not provide summary data for breast cancer-specific mortality risk [21]. Moreover, it remains unknown whether type of alcoholic beverages consumed plays a role [6, 7, 9, 19].

Here we report results from a mortality analysis for a cohort of women with invasive breast cancer, who participated in the Women’s Contraceptive and Reproductive Experiences (CARE) Study. The objective of this analysis was to investigate whether risk of dying from breast cancer is associated with pre-diagnosis alcohol consumption overall or with specific type of alcohol beverages consumed (wine, beer, and liquor).

## Methods

### Study population and data collection

The study population comprised breast cancer patients who participated in the Women’s CARE Study, a population-based multi-center breast cancer case-control study. Methods used in conducting the study were reported previously [22]. In brief, 4575 (1622 black and 2953 white) women aged 35 to 64 years when diagnosed with histologically confirmed first primary invasive breast cancer (International Classification of Diseases for Oncology (ICD-O) codes C50.0-C50.9) were recruited at five field sites (Atlanta, Detroit, Los Angeles, Philadelphia, and Seattle) between July 1994 and April 1998. The Women’s CARE Study protocol was approved by the institutional review boards at all participating institutions.

Information on exposures occurring before breast cancer diagnosis was collected shortly after case patients’ breast cancer diagnoses (average: 5.1 months) by trained staff who administered standardized in-person interviews using a structured questionnaire. The questionnaire covered demographic characteristics, alcohol consumption, medical and reproductive history, oral contraceptive use, menopausal hormonal therapy use, mammographic screening patterns, lifetime exercise participation, and smoking history. Tumor characteristics, including tumor stage at diagnosis and estrogen receptor (ER) status, were abstracted from medical records in Philadelphia and from Surveillance, Epidemiology and End Results (SEER) registry records at the other study sites.

### Assessment of alcohol consumption

A positive history of alcohol consumption prior to breast cancer diagnosis was defined as having consumed at least 12 alcoholic drinks overall and at least one drink a month for 6 or more months. One drink was equivalent to 12 oz. of beer, 4 oz. of wine, or 1.5 oz. of liquor. Women were asked the age at which they first consumed alcohol, the types of alcoholic beverage, the number of drinks for each type of alcohol they consumed per week or per month at that age, and the age at which the reported alcohol consumption pattern changed. Age at which drinking pattern changed marked the end of the first drinking interval and the start of the second. Additional intervals were recorded for each change reported. Consumption was recorded up to the patient’s date of diagnosis. We calculated the number of drinks consumed per week for each year of age, for each beverage (wine, beer, or liquor), and for all beverages combined.

The alcohol consumption variables defined for this analysis included: drinking status (non-drinkers, drinkers) and average weekly alcohol consumption from age 15 years until breast cancer diagnosis (non-drinkers, < 1, 1–< 3, 3–< 7, and ≥ 7 drinks per week), and two time periods of recent consumption before breast cancer diagnosis (non-drinkers, < 1, 1–< 3, 3–< 7, ≥7 drinks per week, and “drinkers who did not drink in this time period”). Recent consumption analyses assessed average alcohol intake in two mutually exclusive time periods: 1) the 5-year period beginning 7 years before breast cancer diagnosis and ending 2 years before diagnosis (i.e., excluding the two years before breast cancer diagnosis to avoid any disease-related changes in alcohol consumption that might have occurred, herein referred to as “recent 5-year period”), and 2) the 2-year period beginning 2 years prior to diagnosis and ending when breast cancer was diagnosed (herein referred to as “recent 2-year period”). In our analyses by beverage type, consumption categories were: non-drinkers, < 1, 1–< 3, and ≥ 3 drinks per week from age 15 years until breast cancer diagnosis.

### Vital status follow-up

As described previously [23], women were followed annually (through December 2004 in Atlanta, Detroit and Seattle, through December 2005 in Philadelphia, and through December 2007 in Los Angeles) to determine vital status, and if death occurred, date of death and cause of death were recorded. The Philadelphia field site used state death records to track vital status. The other study sites used standard SEER follow-up procedures. During follow up, 1068 (528 black, 540 white) women died of all causes and 832 (414 blacks, 418 whites) died from breast cancer.

### Statistical analyses

Multivariable Cox proportional hazards regression models were fit to data and provided adjusted hazard ratios (HRs) and 95% confidence intervals (CIs) for the associations of pre-diagnosis alcohol consumption variables with breast cancer-specific mortality (ICD codes ICD9–174, ICD10-C50) [24], with mortality due to causes other than breast cancer, and with all-cause mortality. The time scale for analysis beginning at breast cancer diagnosis was age in days extending to death or to end of follow-up. When the outcome of interest was breast cancer-specific mortality, women who died from other causes were censored on their dates of death. When the outcome of interest was mortality due to causes other than breast cancer, women who died from breast cancer were censored on their dates of death.

Our statistical models were stratified by age in years at diagnosis, and adjusted for study site (Atlanta, Detroit, Los Angeles, Philadelphia, or Seattle), race (black, white), education (less than high school, high school, technical school or some college, college graduate), household income (0–< 2, 2–< 3, 3–< 5, 5–< 7, ≥7 times the federal poverty guideline for 1996 [25], where “1996” is the approximate midpoint of the diagnosis years for case-patient participants in the Women’s CARE Study), number of mammogram visits during the 5 years before breast cancer diagnosis (0, 1, 2–3, ≥4), body mass index (BMI) 5-years before diagnosis (< 20, 20–24.9, 25–29.9, ≥30 kg/m^2^), number of comorbidities diagnosed before breast cancer diagnosis (0, 1, ≥2 based on diagnoses of hypertension, myocardial infarction, stroke, diabetes, and cancers other than non-melanoma skin cancers), smoking status (never, former, current smoker), tumor stage (localized, non-localized), estrogen receptor (ER) status (ER positive, ER+; ER negative, ER–; unknown), and histologic type of breast cancer (ductal, lobular, other). In analyses for a specific type of alcohol, our models additionally adjusted for other types of alcohol (wine adjusted for beer and liquor, beer adjusted for wine and liquor, liquor adjusted for beer and wine).

Other potential confounders, including first-degree family history of breast cancer, age at menarche, number of full-term pregnancies, menopausal status, menopausal hormone therapy use, average MET-hours per week of physical activity, tumor size, and tumor grade, had minimal influence on estimated hazard ratios and hence were not included in the final statistical models.

Tests for trend were conducted by fitting the median value in each exposure category and testing whether the slope coefficient differed from zero. Likelihood ratio tests were conducted to explore effect modifiers. The potential effect modifiers of interest were: household income (< 3 times vs. ≥3 times the federal poverty guideline), race (black women vs. white women), education (≤ high school vs. >high school), menopausal status at diagnosis (premenopausal vs. postmenopausal), BMI 5-years before diagnosis (< 25 vs. ≥25 kg/m^2^), comorbid conditions (no vs. yes), cigarette smoking status (never vs. ever), stage of breast cancer at diagnosis (localized vs. non-localized), ER status of the tumor (positive vs. negative), and histologic type (ductal vs. lobular).

We excluded women from the analytic cohort who had unknown values for a variable when the unknown category comprised fewer than 0.5% of the participants: 22 women with incomplete information on alcohol consumption, 22 women missing information on BMI 5-years before diagnosis, 7 women with unknown number of mammograms within the 5 years before breast cancer diagnosis, and 1 woman missing information on education. Thus, 4523 case-patients (1598 blacks and 2925 whites) comprised the analytic cohort. Among these women, 1055 (519 blacks, 536 whites) died during follow up (median, 8.6 years), including 824 (409 blacks, 415 whites) who died from breast cancer.

## Results

### Characteristics

The mean age at breast cancer diagnosis was 49.7 years among these women who were, by design, only eligible for the Women’s CARE Study if they had been diagnosed at ages 35 to 64 years. Compared to non-drinkers, drinkers, who had ever drunk alcohol from age 15 years until breast cancer diagnosis, were more likely to be younger, premenopausal, living in Seattle, white, more educated, former or current smokers, and comorbidity-free, and to have lower BMI and higher household income levels (All *P* ≤ 0.008, Table 1). They were also more likely to have been diagnosed with a localized, ER+ tumor (Both *P* ≤ 0 .001). Drinkers did not differ from non-drinkers on number of mammograms in the 5 years before diagnosis (*P* = 0.83) or histologic type of breast cancer (*P* = 0.24).

**Table 1:** Frequency distribution (%^a^) of 4523 women with invasive breast cancer by selected characteristics at diagnosis and alcohol drinking status from age 15 years until diagnosis

	Non-drinkers (*N* = 1779)	Drinkers (*N* = 2744)	*P* value^b^
Age at diagnosis (years)			< 0.0001
35–44	515 (29.0)	914 (33.3)	
45–54	617 (34.7)	994 (36.2)	
55–64	647 (36.4)	836 (30.5)	
Study site			< 0.0001
Atlanta	331 (18.6)	545 (19.9)	
Seattle	285 (16.0)	767 (28.0)	
Detroit	299 (16.8)	367 (13.4)	
Philadelphia	306 (17.2)	393 (14.3)	
Los Angeles	558 (31.4)	672 (24.5)	
Race			< 0.0001
White	949 (53.3)	1976 (72.0)	
Black	830 (46.7)	768 (28.0)	
Education			< 0.0001
<High school	177 (10.0)	214 (7.8)	
High School	620 (34.9)	703 (25.6)	
Technical school/some college	597 (33.6)	867 (31.6)	
College graduates	385 (21.6)	960 (35.0)	
Household income, times the federal poverty guideline			0.008
0–< 2	313 (17.6)	450 (16.4)	
2–< 3	299 (16.8)	456 (16.6)	
3–< 5	453 (25.5)	603 (22.0)	
5–< 7	391 (22.0)	625 (22.8)	
≥7	279 (15.7)	530 (19.3)	
Unknown	44 (2.5)	80 (2.9)	
Menopausal status at diagnosis			< 0.0001
Premenopausal	742 (41.7)	1353 (49.3)	
Postmenopausal	835 (46.9)	1066 (38.9)	
Unknown	202 (11.4)	325 (11.8)	
Number of mammograms in 5 years before diagnosis			0.83
0	536 (30.1)	809 (29.5)	
1	313 (17.6)	477 (17.4)	
2–3	396 (22.3)	643 (23.4)	
≥4	534 (30.0)	815 (29.7)	
Body mass index 5-yrs before diagnosis (kg/m^2^)			< 0.0001
< 20	150 (8.4)	334 (12.2)	
20–24.9	698 (39.2)	1371 (50.0)	
25–29.9	505 (28.4)	678 (24.7)	
≥30	426 (24.0)	361 (13.2)	
Number of comorbid conditions^c^			< 0.0001
0	1032 (58.0)	1809 (65.9)	
1	576 (32.4)	767 (28.0)	
≥2	171 (9.6)	168 (6.1)	
Cigarette smoking status at diagnosis			< 0.0001
Never	1078 (60.6)	996 (36.3)	
Former	426 (24.0)	1060 (38.6)	
Current	275 (15.5)	688 (25.1)	
Stage of disease at diagnosis			0.001
Localized	1022 (57.5)	1710 (62.3)	
Non-localized	757 (42.5)	1034 (37.7)	
Estrogen receptor status of tumor			< 0.0001
Positive	969 (54.5)	1685 (61.4)	
Negative	558 (31.4)	743 (27.1)	
Unknown	252 (14.2)	316 (11.5)	
Histologic type			0.24
Ductal	1348 (75.8)	2071 (75.5)	
Lobular	193 (10.9)	336 (12.2)	
Others	238 (13.4)	337 (12.3)	

^a^Percentage may not sum to 100% due to rounding

^b^*P*-value ascertained from Chi-square test,

^c^Comorbidities included hypertension, myocardial infarction, stroke, diabetes, and cancers other than non-melanoma skin cancers

### Alcohol consumption and mortality risk

Ever drinking alcohol from age 15 years until breast cancer diagnosis was associated with a modest decrease in risk of breast cancer-specific mortality (HR = 0.87, 95% CI = 0.75–1.01), although the 95% CI included 1.0 (Table 2). Average weekly alcohol consumption from age 15 years until breast cancer diagnosis was inversely associated with breast cancer-specific mortality risk (*P*_trend_ = 0.01). Compared to non-drinkers, women who averaged at least 7 drinks of alcohol per week from age 15 years until breast cancer diagnosis had a modest reduction in risk of breast cancer-specific mortality (HR = 0.75, 95% CI = 0.56–1.00). Similar risk patterns for breast cancer-specific mortality were observed for alcohol consumption in the recent 5-year period ending 2 years prior to diagnosis and in the most recent 2-year period prior to breast cancer diagnosis; however, the corresponding 95% CIs of HRs associated with the highest category of average weekly alcohol consumption during these two mutually exclusive recent time periods excluded 1.0 (recent 5-year period ending 2 years before diagnosis: HR = 0.74, 95% CI = 0.57–0.95; recent 2-year period before diagnosis: HR = 0.73, 95% CI = 0.56–0.95). No association with ever drinking alcohol or with average weekly alcohol consumption drinking during different time periods was observed for risk of mortality due to causes other than breast cancer. The inverse associations of these alcohol consumption variables with risk of all-cause mortality were similar to those with risk of breast cancer-specific mortality.

**Table 2:** Multivariable adjusted^a^ HR and 95% CI for mortality risk associated with alcohol consumption before breast cancer diagnosis

	Person-years	Breast cancer-specific mortality		Mortality due to causes other than breast cancer		All-cause mortality	
		No. Death	HR (95% CI)	No. Death	HR (95% CI)	No. Death	HR (95% CI)
Alcohol drinking status prior to breast cancer diagnosis							
Non-drinkers	14,468	382	Referent	87	Referent	469	Referent
Drinkers	22,684	442	0.87 (0.75–1.01)	144	1.17 (0.87–1.56)	586	0.93 (0.81–1.06)
Average alcohol consumption from age 15 years until breast cancer diagnosis (drinks/week)							
Non-drinkers	14,468	382	Referent	87	Referent	469	Referent
< 1	6555	140	1.04 (0.85–1.27)	34	1.21 (0.80–1.84)	174	1.07 (0.90–1.28)
1–< 3	7485	139	0.82 (0.67–1.01)	45	1.28 (0.87–1.87)	184	0.90 (0.76–1.08)
3–< 7	5490	106	0.80 (0.64–1.01)	41	1.17 (0.79–1.74)	147	0.88 (0.72–1.07)
≥ 7	3153	57	0.75 (0.56–1.00)	24	0.95 (0.59–1.54)	81	0.79 (0.62–1.01)
*P*_*t*rend_			*0.01*		*0.73*		*0.03*
Average alcohol consumption in recent 5-year period beginning 7 years before breast cancer diagnosis and continuing until 2 years before breast cancer diagnosis (drinks/week)							
Non-drinkers	14,468	382	Referent	87	Referent	469	Referent
< 1	4385	85	0.88 (0.69–1.12)	24	1.27 (0.80–2.04)	109	0.94 (0.76–1.17)
1–< 3	4835	82	0.83 (0.65–1.07)	27	1.28 (0.82–2.02)	109	0.91 (0.74–1.13)
3–< 7	4464	91	0.87 (0.68–1.10)	24	1.02 (0.63–1.63)	115	0.90 (0.73–1.11)
≥ 7	4614	78	0.74 (0.57–0.95)	27	0.89 (0.56–1.40)	105	0.77 (0.62–0.97)
*P*_trend_			*0.04*		*0.50*		*0.05*
Drank alcohol, but not in this period	4386	106	1.01 (0.81–1.26)	42	1.37 (0.92–2.02)	148	1.08 (0.89–1.31)
Average alcohol consumption in recent 2-year period beginning 2 years before breast cancer diagnosis until breast cancer diagnosis (drinks/week)							
Non-drinkers	14,468	382	Referent	87	Referent	469	Referent
< 1	3863	67	0.76 (0.58–1.00)	19	1.21 (0.72–2.02)	86	0.84 (0.66–1.06)
1–< 3	4560	79	0.82 (0.64–1.06)	30	1.53 (0.99–2.36)	109	0.94 (0.76–1.17)
3–< 7	4000	79	0.86 (0.67–1.11)	19	0.90 (0.54–1.52)	98	0.87 (0.69–1.09)
≥ 7	4418	74	0.73 (0.56–0.95)	27	0.97 (0.61–1.54)	101	0.79 (0.63–0.99)
*P*_trend_			*0.08*		*0.60*		*0.12*
Drank alcohol, but not in this period	5842	143	1.06 (0.87–1.30)	49	1.21 (0.84–1.76)	192	1.09 (0.91–1.30)

*Abbreviations*: *HR* hazard ratio, *CI* confidence interval

^a^All models are stratified by age at diagnosis, and include study site, race, education, household income, number of mammograms within the 5 years before breast cancer diagnosis, body mass index 5-years before diagnosis, number of comorbidities before diagnosis, smoking history, stage, estrogen receptor status, and histologic type of breast cancer tumor

### Wine, beer, or liquor consumption and mortality risk

Analyses by beverage type showed that wine consumption was inversely associated with breast cancer-specific mortality risk (wine *P*_trend_ = 0.06, beer *P*_trend_ = 0.24, liquor *P*_trend_ = 0.74; Table 3). Compared to non-drinking women, those who consumed, on average, at least 3 drinks of wine per week from age 15 years until breast cancer diagnosis had a modest reduction in breast cancer-specific mortality risk (HR = 0.76, 95% CI = 0.53–1.11). Similar risk reduction associated with the highest level of wine consumption was observed for all-cause mortality (HR = 0.73, 95% CI = 0.53–1.01). Although we did not observe statistically significant trends in risk overall for either beer or liquor consumption, the highest level of beer consumption (≥ 3 drinks/week) was modestly associated with breast cancer-specific and all-cause mortality, which was similar to the findings for wine (breast cancer-specific: HR = 0.79, 95% CI = 0.59–1.07; all-cause: HR = 0.77, 95% CI = 0.59–1.00). No association with any specific type of alcohol consumption was observed for risk of mortality due to causes other than breast cancer.

**Table 3:** Multivariable adjusted^a^ HR and 95% CI for mortality risk associated with wine, beer, or liquor consumption from age 15 years until breast cancer diagnosis

	Person-years	Breast cancer-specific mortality		Mortality due to causes other than breast cancer		All-cause mortality	
		No. Death	HR (95% CI)	No. Death	HR (95% CI)	No. Death	HR (95% CI)
Non-drinkers	14,468	382	Referent	87	Referent	469	Referent
Wine, average drinks/week							
< 1	9353	191	0.97 (0.78–1.19)	43	0.83 (0.56–1.23)	234	0.92 (0.77–1.11)
1–< 3	5106	80	0.78 (0.60–1.03)	30	1.04 (0.67–1.61)	110	0.83 (0.66–1.04)
≥ 3	2560	35	0.76 (0.53–1.11)	9	0.58 (0.29–1.20)	44	0.73 (0.53–1.01)
*P*_*t*rend_			*0.06*		*0.27*		*0.04*
Beer, average drinks/week							
< 1	7652	143	0.82 (0.66–1.02)	47	1.37 (0.93–2.02)	190	0.91 (0.75–1.10)
1–< 3	3706	71	0.94 (0.72–1.23)	29	1.24 (0.79–1.95)	100	0.99 (0.79–1.25)
≥ 3	2471	57	0.79 (0.59–1.07)	17	0.73 (0.42–1.27)	74	0.77 (0.59–1.00)
*P*_*t*rend_			*0.24*		*0.19*		*0.08*
Liquor, average drinks/week							
< 1	10,978	218	1.09 (0.88–1.34)	54	0.99 (0.67–1.46)	272	1.08 (0.90–1.30)
1–< 3	4330	84	0.97 (0.74–1.27)	35	1.17 (0.75–1.82)	119	1.03 (0.82–1.30)
≥ 3	2572	54	0.99 (0.72–1.37)	25	1.07 (0.65–1.74)	79	1.04 (0.80–1.35)
*P*_*t*rend_			*0.74*		*0.14*		*0.98*

*Abbreviations*: *HR* hazard ratio, *CI* confidence interval

^a^All models are stratified by age at diagnosis, and include study site, race, education, household income, number of mammograms within the 5 years before breast cancer diagnosis, body mass index 5-years before diagnosis, number of comorbidities before breast cancer diagnosis, smoking history, stage, estrogen receptor status, histologic type of breast cancer tumor, and consumption of other types of alcohol using categories of drinks/week (wine adjusted for beer and liquor, beer adjusted for wine and liquor, liquor adjusted for beer and wine)

### Exploratory analyses for potential effect modifiers of the association between specific type of alcohol consumed and breast cancer-specific mortality risk

We conducted exploratory effect modification analyses to determine whether the mortality association with wine, beer, or liquor consumption differed among subgroups of potential effect modifiers of interest; we found that the observed inverse association between average weekly wine consumption from age 15 years until breast cancer diagnosis and risk of breast cancer-specific mortality was modified by household income level (Table 4). Among women with a lower household income (< 3 times the federal poverty guideline), women in the highest category of average weekly wine consumption (≥3 drinks/week) from age 15 years until breast cancer diagnosis had 68% lower risk of breast cancer-specific mortality than non-drinkers (HR = 0.32, 95% CI = 0.14–0.74, *P*_trend_ = 0.003); no reduction in risk was observed among those with a higher household income (≥3 times the federal poverty guideline, HR = 1.09, 95% CI = 0.72–1.63, *P*_trend_ = 0.98, likelihood ratio test for heterogeneity of trends for a lower vs. higher household income: *P*_heterogeneity_ = 0.005). No effect modification was observed for beer or liquor (results not shown).

**Table 4:** Potential effect modifiers of multivariable adjusted^a^ HR and 95% CI for risk of breast cancer-specific mortality associated with wine consumption from age 15 years until breast cancer diagnosis

		Wine consumption from age 15 years until breast cancer diagnosis, average drinks/week					
		Non-drinkers	< 1	1–< 3	≥3	*P*_trend_	*P*_heterogeneity_
Household income, times the federal poverty guideline							
< 3	No. Death	148	67	27	6		
	HR (95% CI)	Referent	0.94 (0.69–1.28)	0.70 (0.46–1.07)	0.32 (0.14–0.74)	*0.003*	*0.005*
≥ 3	No. Death	226	117	50	29		
	HR (95% CI)	Referent	0.96 (0.74–1.23)	0.82 (0.59–1.14)	1.09 (0.72–1.63)	*0.98*	
Race							
Black	No. Death	215	75	26	9		
	HR (95% CI)	Referent	1.06 (0.79–1.41)	0.64 (0.42–0.98)	0.65 (0.33–1.30)	*0.05*	*0.23*
White	No. Death	167	116	54	26		
	HR (95% CI)	Referent	0.92 (0.71–1.19)	0.88 (0.64–1.22)	0.82 (0.53–1.26)	*0.33*	
Education							
≤ High school	No. Death	185	59	27	13		
	HR (95% CI)	Referent	0.82 (0.60–1.12)	0.88 (0.58–1.35)	1.09 (0.61–1.96)	*0.93*	*0.11*
> High school	No. Death	197	132	53	22		
	HR (95% CI)	Referent	1.03 (0.80–1.31)	0.73 (0.52–1.00)	0.64 (0.40–1.00)	*0.02*	
Menopausal status at diagnosis							
Pre-MP	No. Death	177	109	49	19		
	HR (95% CI)	Referent	0.95 (0.73–1.24)	0.95 (0.68–1.34)	0.73 (0.44–1.19)	*0.24*	*0.42*
Post-MP	No. Death	166	61	21	11		
	HR (95% CI)	Referent	0.93 (0.68–1.28)	0.68 (0.43–1.09)	0.63 (0.34–1.19)	*0.07*	
BMI 5-years before diagnosis (kg/m^2^)							
< 25	No. Death	154	113	53	23		
	HR (95% CI)	Referent	0.97 (0.75–1.25)	0.79 (0.57–1.10)	0.78 (0.49–1.22)	*0.15*	*0.80*
≥ 25	No. Death	228	78	27	12		
	HR (95% CI)	Referent	0.96 (0.72–1.27)	0.75 (0.49–1.14)	0.73 (0.40–1.33)	*0.17*	
Comorbid condition^b^							
No	No. Death	206	125	53	27		
	HR (95% CI)	Referent	0.92 (0.72–1.18)	0.80 (0.58–1.11)	0.74 (0.48–1.12)	*0.10*	*0.96*
Yes	No. Death	176	66	27	8		
	HR (95% CI)	Referent	1.04 (0.77–1.41)	0.74 (0.49–1.13)	0.84 (0.41–1.75)	*0.30*	
Cigarette smoking							
Never	No. Death	227	84	30	10		
	HR (95% CI)	Referent	1.06 (0.79–1.41)	1.00 (0.66–1.51)	0.69 (0.36–1.33)	*0.34*	*0.77*
Ever	No. Death	155	107	50	25		
	HR (95% CI)	Referent	0.91 (0.70–1.17)	0.68 (0.49–0.94)	0.79 (0.51–1.22)	*0.08*	
Stage of breast cancer at diagnosis							
Localized	No. Death	100	55	27	11		
	HR (95% CI)	Referent	1.10 (0.78–1.54)	1.06 (0.69–1.65)	0.80 (0.42–1.51)	*0.60*	*0.43*
Non-localized	No. Death	282	136	53	24		
	HR (95% CI)	Referent	0.92 (0.73–1.16)	0.69 (0.50–0.94)	0.76 (0.49–1.17)	*0.05*	
Estrogen receptor status							
Positive	No. Death	161	82	40	20		
	HR (95% CI)	Referent	0.93 (0.70–1.24)	0.80 (0.55–1.16)	0.87 (0.53–1.41)	*0.35*	*0.80*
Negative	No. Death	160	93	35	13		
	HR (95% CI)	Referent	1.11 (0.84–1.49)	0.87 (0.59–1.29)	0.76 (0.43–1.37)	*0.25*	
Histogologic type							
Ductal	No. Death	286	150	62	24		
	HR (95% CI)	Referent	0.97 (0.77–1.22)	0.74 (0.54–1.00)	0.66 (0.43–1.03)	*0.02*	*0.33*
Lobular	No. Death	37	25	6	8		
	HR (95% CI)	Referent	1.17 (0.72–1.93)	0.50 (0.21–1.19)	1.21 (0.56–2.61)	*0.91*	

*Abbreviations*: *HR* hazard ratio, *CI* confidence interval, *MP* menopausal

^a^All models are stratified by age at diagnosis, and include study site, race, education, household income, number of mammograms within the 5 years before breast cancer diagnosis, body mass index 5-years before diagnosis, number of comorbidities before breast cancer diagnosis, smoking history, stage, estrogen receptor status, histologic type of breast cancer tumor, beer consumption, and liquor consumption

^b^Comorbidities included hypertension, myocardial infarction, stroke, diabetes, and cancers other than non-melanoma skin cancers

## Discussion

In this large cohort of women diagnosed with invasive breast cancer between the ages of 35 and 64 years, those who drank, on average, at least seven alcoholic beverages per week from age 15 years until breast cancer diagnosis had a 25% non-statistically significant lower risk of breast cancer-specific mortality than non-drinkers of alcohol. Similar magnitudes of risk reduction were observed for alcohol consumption in the recent 5-year period ending 2 years before diagnosis and in the most recent 2-year period before breast cancer diagnosis. Analyses by beverage type suggested that wine consumption was inversely associated with risk of breast cancer-specific mortality.

Previous findings for the association between pre-diagnosis alcohol consumption and risk of breast cancer-specific mortality are inconsistent [6–20], which could be due, at least partly, to variations in statistical power, time periods of alcohol consumption, or levels of alcohol consumption in these studies. Reding et al. [6] report that in 1286 women diagnosed with invasive breast cancer at age 45 years or younger (364 deaths, 335 from breast cancer), long-term alcohol consumption (from age 15 years until breast cancer diagnosis) and alcohol consumption in a recent 5-year time period were associated with a decreased risk of death from breast cancer. We used the same definitions for long-term alcohol consumption and recent consumption as were used by Reding et al. and replicated their findings. Lowry et al. [8] present findings from the Women’s Health Initiative (WHI) observational study which are consistent with our finding that pre-diagnosis alcohol consumption is inversely associated with breast cancer-specific mortality risk. Newcomb et al. [7] found an inverse association of breast cancer-specific mortality that was limited to women in the moderate category of alcohol consumption (3–6 drinks/week) relative to non-drinkers in the Collaborative Breast Cancer Study (CBCS) and observed no association in heavier drinkers (≥10 drinks/week). This suggests a U-shaped relationship between lifetime alcohol consumption and breast cancer specific mortality. Three studies report an increased risk of breast cancer-specific mortality, associated with higher daily alcohol consumption (e.g., a 6% increase in risk, 95% CI = 3–10% with > 20 g/day of alcohol consumed) [9–11]. Our data provide no evidence that alcohol consumption before breast cancer diagnosis increases subsequent risk of death from breast cancer. It is possible that the relatively low levels of alcohol consumed in our study participants (95th percentile among drinkers was 12.9 drinks per week) may have limited our ability to detect this association.

Only four published epidemiologic studies provide data regarding whether the impact of alcohol consumption on breast cancer death varies by type of alcohol [6, 7, 9, 19]. Reding et al. [6] report that wine consumed in the five years before diagnosis was associated with a decreased risk of breast cancer-specific mortality, but neither beer nor liquor consumed in that period was associated with breast cancer-specific mortality risk. Newcomb et al. [7] report that the association between moderate lifetime pre-diagnosis alcohol consumption (3–6 drinks/week) and decreased risk of breast cancer-specific mortality in the CBCS did not vary by type of alcoholic beverage. Jain et al. [9] observed a 15% increase in breast cancer-specific mortality risk associated with daily consumption of more than 10 g of wine (HR = 1.146, 95% CI = 1.111–1.182) and a 5% decrease in risk associated with daily consumption of more than 10 g of spirits (HR = 0.945, 95% CI = 0.915–0.976). Consumption of more than 10 g/day of beer was not associated with breast cancer-specific mortality (HR = 1.025, 95% CI = 0.969–1.085). Din et al. [19] report that overall, breast cancer-specific mortality risk was not associated with the type of alcohol consumed before diagnosis, whereas they observed statistically significant associations in analyses stratified by stage of breast cancer at diagnosis, including a decreased risk of death due to breast cancer associated with low wine intake (0.75–3.75 drinks/week) among women diagnosed with localized disease and increased risk of breast cancer-specific death associated with high wine intake (10.00–36.00 drinks/week) among those with regional or distant disease. In our analyses, we did not find clear evidence that the disease stage at breast cancer diagnosis modifies the association between wine intake and risk of breast cancer-specific mortality. In general, our results support those reported by Reding et al., which showed that wine consumption before breast cancer diagnosis is associated with lower risk of breast cancer-specific mortality.

Alcohol consumption has been linked to increased risk of developing breast cancer [1, 4, 5], possibly because ethanol increases estrogen levels, inducing DNA damage, and interfering with DNA repair [26–28]. Thus, it is plausible to hypothesize that alcohol consumption prior to diagnosis would have an adverse impact on tumor progression and breast cancer recurrence. McCarty CA et al. report that the impact of alcohol consumption on breast cancer risk varies by genotype(s), which are involved in alcohol-metabolizing pathways [29]. For example, they found that alcohol consumption was positively associated with breast cancer risk among women with the GG allele of alcohol dehydrogenase 1B (ADH1B) gene, but appeared to be inversely associated with risk among women with the GA or AA allele. Moreover, many compounds other than ethanol are present in different types of alcoholic beverages and the effects on health outcomes may differ. The association between wine and decreased mortality risk may be due to wine’s high antioxidant levels [30] or to beneficial effects of other compounds such as resveratrol in red wine [31]. Bioactive constituents in wine (e.g. polyphenols) have been hypothesized to reduce the risk of death after cancer [32, 33]. Quercetin, a flavonoid abundantly present in red wine has also been shown to inhibit tumor growth and increase survival in animal studies [34].

A major strength of our study is the number of breast cancer deaths, which is greater than those in all previous individual studies on this topic except the CBCS [7]. We collected information on pre-diagnosis alcohol consumption from age 15 until the date of diagnosis, whereas most previous studies collected alcohol consumption for only one time point. We also collected detailed information on potential risk factors for breast cancer incidence and mortality, which enabled us to assess these as potential confounders and effect modifiers. Moreover, our study is one of only a few that have investigated the mortality associations with type of alcohol consumed [6, 7, 9, 19].

This study has several limitations. First, we used self-reported alcohol consumption, which may be inaccurate. Such measurement error, however, would be expected to be non-differential with respect to mortality, resulting in attenuation of the true underlying association. Second, the Women’s CARE Study questionnaire was designed to assess etiologic risk factors for breast cancer and did not collect information on alcohol consumption after diagnosis. While alcohol consumption patterns may change over time, several studies have shown that alcohol consumption does not change following a breast cancer diagnosis [7, 17, 35, 36]. The CBCS [7] and the WHI [8] investigated the impact of alcohol consumption before and after breast cancer diagnosis on mortality risk, finding that alcohol consumption before diagnosis was associated with decreased risk of breast cancer-specific mortality (details described above), but consumption after diagnosis was not. Moreover, in a pooled analysis of 9329 breast cancer patients [37], a meta-analysis of 11 published studies [21], and a collaborative analysis of 29,239 breast cancer patients [21], no clear evidence was observed that post-diagnosis alcohol consumption was associated with breast cancer-specific mortality risk. Third, we do not have medical record data on treatment; however, by controlling for age, stage of disease and hormone receptor status, we have accounted for most determinants of treatment, although residual confounding may still exist. Fourth, a comparison of alcohol drinkers to non-drinkers in our study showed that drinkers tended to be younger, premenopausal, white, former or current smokers, and without comorbidities, who had higher education and household income levels and lower BMI. They were also more likely to have localized disease or an ER+ tumor. Some of these factors, such as higher education and household income levels, lack of comorbidities, localized stage at diagnosis, and an ER+ tumor, may be associated with decreased risk of breast cancer-specific mortality. Despite adjusting for these factors in our statistical models, we are unable to rule out residual confounding as the explanation for our results, especially for the observed protective effect of wine consumption. Fifth, a small number of women (*n* = 231) died of causes other than breast cancer limiting our statistical power to assess the effects of alcohol consumption on other specific causes of death, such as heart disease. Finally, because we lack genotype data, we are unable to determine whether the observed inverse association between alcohol consumption (particularly wine consumption) and risk of breast cancer-specific mortality is modified by genotypic variation (e.g., ADH1B).

## Conclusions

Overall, we found no evidence that alcohol consumed over a woman’s life before her breast cancer diagnosis increases her subsequent risk of death from breast cancer. Future studies that incorporate information on types of alcohol consumed before diagnosis, during treatment (if any), and after treatment, are warranted to clarify the somewhat differing results of studies to date.

## Data Availability

The data supporting the conclusions of this report are included within the article.

## Abbreviations

BMI: Body mass index
CARE: Contraceptive and Reproductive Experiences
CI: Confidence interval
ER: Estrogen receptor
HR: Hazard ratio
ICD-O: International Classification of Diseases for Oncology
SEER: Surveillance, Epidemiology and End Results
